# Prediction Models for the Clinical Severity of Patients With COVID-19 in Korea: Retrospective Multicenter Cohort Study

**DOI:** 10.2196/25852

**Published:** 2021-04-16

**Authors:** Bumjo Oh, Suhyun Hwangbo, Taeyeong Jung, Kyungha Min, Chanhee Lee, Catherine Apio, Hyejin Lee, Seungyeoun Lee, Min Kyong Moon, Shin-Woo Kim, Taesung Park

**Affiliations:** 1 Department of Family Medicine Seoul Metropolitan Government Seoul National University Boramae Medical Center Seoul Republic of Korea; 2 Interdisciplinary Program in Bioinformatics Seoul National University Seoul Republic of Korea; 3 Department of Family Medicine Seoul National University Bundang Hospital Gyeonggi-do Republic of Korea; 4 Department of Mathematics and Statistics Sejong University Seoul Republic of Korea; 5 Department of Internal Medicine Seoul Metropolitan Government Seoul National University Boramae Medical Center Seoul Republic of Korea; 6 Department of Internal Medicine Kyungpook National University Daegu Republic of Korea; 7 Department of Statistics Seoul National University Seoul Republic of Korea

**Keywords:** clinical decision support system, clinical characteristics, COVID-19, SARS-CoV-2, prognostic tool, severity

## Abstract

**Background:**

Limited information is available about the present characteristics and dynamic clinical changes that occur in patients with COVID-19 during the early phase of the illness.

**Objective:**

This study aimed to develop and validate machine learning models based on clinical features to assess the risk of severe disease and triage for COVID-19 patients upon hospital admission.

**Methods:**

This retrospective multicenter cohort study included patients with COVID-19 who were released from quarantine until April 30, 2020, in Korea. A total of 5628 patients were included in the training and testing cohorts to train and validate the models that predict clinical severity and the duration of hospitalization, and the clinical severity score was defined at four levels: mild, moderate, severe, and critical.

**Results:**

Out of a total of 5601 patients, 4455 (79.5%), 330 (5.9%), 512 (9.1%), and 301 (5.4%) were included in the mild, moderate, severe, and critical levels, respectively. As risk factors for predicting critical patients, we selected older age, shortness of breath, a high white blood cell count, low hemoglobin levels, a low lymphocyte count, and a low platelet count. We developed 3 prediction models to classify clinical severity levels. For example, the prediction model with 6 variables yielded a predictive power of >0.93 for the area under the receiver operating characteristic curve. We developed a web-based nomogram, using these models.

**Conclusions:**

Our prediction models, along with the web-based nomogram, are expected to be useful for the assessment of the onset of severe and critical illness among patients with COVID-19 and triage patients upon hospital admission.

## Introduction

COVID-19, an infectious disease, is currently spreading at an unprecedented pace. The World Health Organization declared COVID-19 a public health emergency of worldwide concern on January 30, 2020, and subsequently a pandemic on March 11, 2020. The COVID-19 pandemic has posed challenges to public health systems worldwide [1,2].

The clinical spectrum of SARS-CoV-2 infection ranges from asymptomatic to fatal, requiring mechanical ventilation [3]. According to initial data from China, the clinical spectrum of COVID-19 is broad, with most infected individuals experiencing only mild or subclinical illnesses, especially in the early phase of the disease [4]. However, a recent study reported that approximately 14%-30% of hospitalized patients diagnosed with COVID-19 develop a severe respiratory failure that requires intensive care [5-7]. The wide range of outcomes observed, ranging from subpopulations that are mainly asymptomatic to those with substantial fatality rates, calls for risk stratification.

Although dexamethasone and remdesivir have recently been considered a preferred treatment strategy, it is still difficult to use them universally for all patients with COVID-19 [8]; hence, supportive treatments to protect multiorgan functions are a major resource for reducing mortality [9,10]. Several promising innovative drugs and treatment strategies are under investigation; however, until they become commercially available, the capacity of the medical system remains limited, prompting the need for making rationing decisions [10,11]. We argue that early identification of patients at the risk of severe respiratory failure would facilitate better resource planning and help set up effective organizational and clinical interventions, including early pharmacotherapy to prevent admission to the intensive care unit.

Since COVID-19 is a pandemic, many studies have assessed regional clinical features among patients. Pandemic preparedness and strategies differ among countries, and the clinical characteristics of patients admitted to medical facilities seem to vary in different cohorts.

We obtained data on 5628 confirmed patients with COVID-19 admitted to hospitals in Korea and analyzed their clinical features and clinical findings upon admission. Therefore, the objectives of this study are to (1) develop models that predict which individuals are at a high risk of severe disease and their duration of hospitalization in a cohort of hospitalized patients with a confirmed diagnosis of COVID-19 and (b) generate a web-based nomogram based on these models. Our results are expected to provide clinicians with a better understanding of the clinical course of COVID-19 and a guideline for critical care rationing.

## Methods

### Data Source and Study Design

This is a retrospective, multicenter cohort study conducted in Korea. The data used in this study were public data provided by the Korea Disease Control and Prevention Agency (KDCA) in Korea. Data were collected by the KDCA from physicians at multiple centers. The study cohort included 5628 patients with COVID-19 confirmed through the RT–PCR test and hospitalized or released from quarantine upon recovery by April 30, 2020.

A total of 41 variables were recorded for each patient. These 41 variables are classified into 7 types (Multimedia Appendix 1, Table S1). Among the 41 variables provided by KDCA, 35 were used as predictors, including demographics, physical measurements, initial vital signs, comorbidities, and laboratory findings collected upon admission. We excluded 6 pregnancy-related variables because they were applicable only to women. This study was approved by the institutional review board of Seoul National University (protocol# E2008/003-004).

### Definitions of the Primary and Secondary Outcomes

**Figure 1:**
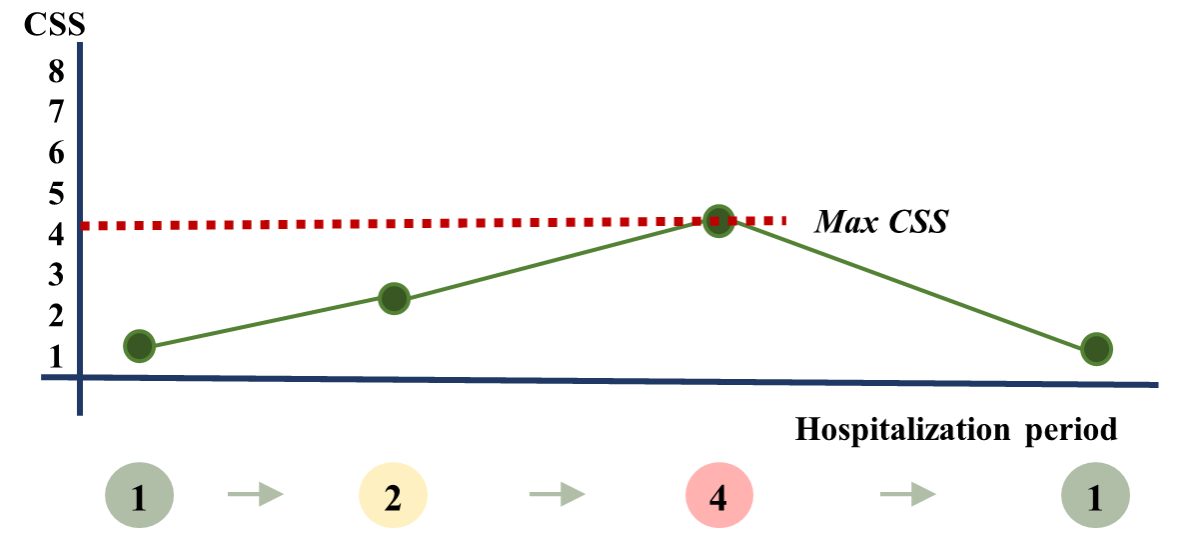
Diagrammatic representation of the definition of the maximum clinical severity score. CSS: clinical severity score.

In this study, the primary outcome of interest is the maximum clinical severity score (CSS). The original CSSs provided by the KDCA have 8 levels (Multimedia Appendix 1, Table S2). The CSSs contain ordered information about the clinical severity of patients with COVID-19. For example, the lowest level (ie, level 1) represents no activity restrictions and the highest level (ie, level 8) represents death. As shown in Figure 1, each patient may go through different CSS levels during the course of hospitalization. For each patient, the “max CSS” was defined as the maximum level of CSS reported through their hospital duration (Figure 1). Instead of the original 8 levels, the severity was reclassified into 4 levels depending on the patient’s condition to determine the appropriate treatment in our study. Accordingly, the modified CSS (mCSS) was defined as mild, moderate, severe, and critical (Multimedia Appendix 1, Table S2). The mild group included patients with no activity restrictions, which corresponded to 1 in the original CSS levels. The moderate group displayed limited activity but did not require oxygen therapy. This group corresponded to the original CSS level of 2. Patients who received oxygen therapy were classified under the severe group and those who received ventilation or extracorporeal membrane oxygenation or those who died were classified under the critical group. The severe group corresponded to original CSS levels of 3 and 4, and the critical group corresponded to original CSS levels of 5, 6, 7, and 8.

The secondary outcome was the total duration of hospitalization from the time of admission to discharge. In Korea, once a patient tests positive for COVID-19 on the RT–PCR test, he/she would be admitted to hospital or an isolation facility immediately. Our data set contains data on only the hospitalized patients with COVID-19 having clinical findings such as blood test results.

### Data Preprocessing

Among 35 predictor variables, 7 variables including body temperature, heart rate, and 5 laboratory results were continuous variables, while all the other variables were categorical variables. Among the 7 continuous variables, body temperature and heart rate were recategorized. Specifically, body temperature was divided into 2 categories with 37.5°C considered the threshold, and the heart rate was divided into 3 groups of <60 beats/min, 60-100 beats/min, and ≥100 beats/min. Among the 28 original categorical variables, age, body mass index (BMI), systolic blood pressure (SBP), and diastolic blood pressure (DBP) were recategorized. Age was originally grouped into 10-year-old intervals: <10 years, 10-19 years, 20-29 years, 30-39 years, 40-49 years, 50-59 years, 60-69 years, 70-79 years, and ≥80 years. Of these groups, the values of the age groups of 0-9 years and 10-19 years were merged into 1 group. For BMI, 5 groups were formed: <18.5 kg/m^2^, 18.5-22.9 kg/m^2^, 23-24.9 kg/m^2^, 25-29.9 kg/m^2^, and ≥30 kg/m^2^. Of these groups, values ranging 25-29.9 kg/m^2^ and ≥30 kg/m^2^ were merged into 1 group. For SBP, 5 groups were initially formed: <120 mmHg, 120-129 mmHg, 130-139 mmHg, 140-159 mmHg, and ≥160 mmHg. For DBP, 4 groups were initially formed: <80 mmHg, 80-89 mmHg, 90-99 mmHg, and ≥100 mmHg. SBP and DBP were divided into 2 groups based on the values of 140 mmHg and 90 mmHg, respectively.

To analyze the primary outcome (ie, mCSS), 5601 samples were used, excluding missing observations. To analyze the secondary outcome (ie, the duration of hospitalization), 5387 samples were used after excluding patients who died through the course of hospitalization. The median duration of hospitalization was 24 days. Accordingly, we classified the duration of hospitalization into two treatment groups: short-term and long-term.

### Predictive Marker Selection Through Univariate Analysis

To identify candidate predictive markers related to the primary and secondary outcomes, univariate analysis was first performed. On univariate analysis, mCSS was considered a continuous variable. We performed correlation analysis between mCSS and continuous predictors using the Pearson, Spearman, and Kendall rank correlation tests [12,13], two-tailed *t* test for binary predictors, and analysis of variance for multilevel categorical predictors. Furthermore, we performed the Cochran–Armitage Trend test [14] to identify predictors with a linear trend of mCSS. For the duration of hospitalization, we used a Cox proportional hazards (CoxPH) model to identify candidate predictive markers [15].

### Development and Evaluation of the Prediction Model

**Figure 2:**
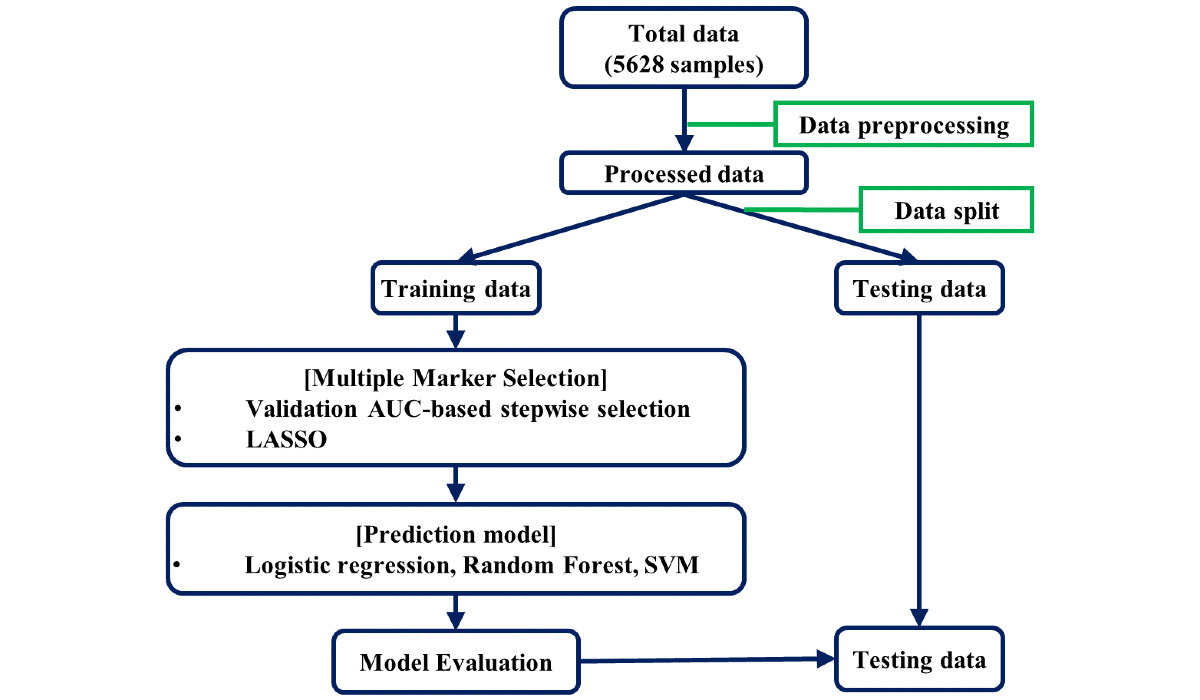
Workflow for model building and evaluation. AUC: area under the curve, LASSO: least absolute shrinkage and selection operator, SVM: support vector machine.

Figure 2 shows the workflow for model development and evaluation. To avoid overfitting, we evaluated testing errors by splitting the total data set into training and testing data sets in a ratio of 2:1 in a stratified manner, by considering the ratio of the max CSS 4 levels and the long- or short-term group. To maintain the same scale for predictor variables, we standardized each predictor variable.

In order to develop models that predict the max CSS, the 4-level mCSS was combined into two levels in three ways such as (1) y_1_: mild (mCSS=1) vs above moderate (mCSS≥2), (2) y_2_: below moderate (mCSS≤2) vs above severe (mCSS≥3), and (3) y_3_: below severe (mCSS≤3) vs critical (mCSS=4). We fit 3 logistic regression models for binary responses. For multiple marker selection, stepwise variable selection was performed on the basis of the area under the receiver operating characteristic curve (AUC) [16], and we used the least absolute shrinkage and selection operator (LASSO) regression method [17,18]. For both stepwise and LASSO variable selections, 5-fold cross-validation was performed. For prediction models, we considered logistic regression, random forest (RF) classification, and a support vector regression machine [19,20]. Each prediction model was fit using markers selected through stepwise and LASSO regression analyses. The performance of each model was evaluated on the basis of the AUC, sensitivity, and specificity. The optimal threshold for sensitivity and specificity was selected as the threshold value with the maximum balanced accuracy. All analyses were implemented in the R package (version 3.6.1, The R Foundation).

## Results

### Demographics and Clinical Characteristics

The demographics and clinical characteristics of the 5628 patients, with particular focus on the risk predictors for mCSS or the duration of hospitalization, are presented in Table 1. A complete list of all predictors is provided in Multimedia Appendix 1, Table S3. Among them, 1785 (31.8%) patients were aged over 60 years, and 2320 (41.2%) were male. In total, 1299 (29.4%) patients were overweight or obese by Asia-Pacific BMI criteria. At the time of initial admission, 1936 (35.3%) patients had an SBP of ≥140 mmHg, and 887 (15.9%) had a body temperature of ≥37.5°C. At the time of diagnosis, the patients experienced the following symptoms: fever (n=1305, 23.2%), sputum production (n=1619, 28.8%), shortness of breath (SOB) (n=666, 11.8%), and altered consciousness or confusion (ACC) (n=35, 0.6%).

The patients had the following underlying comorbidities: diabetes mellitus (DM) (n=691, 12.3%), hypertension (HTN) (n=1201, 21.4%), heart failure (HF) (n=59, 1.0%), asthma (n=128, 2.3%), and chronic obstructive pulmonary disease (COPD) (n=40, 0.7%). Initial mean laboratory values were 13.3 (SD 1.8) g/dL for hemoglobin, 39.2% (SD 5%) for hematocrit, 29.1% (SD 11.7%) for the proportion of lymphocytes, 236,733/µL (SD 82,921/µL) for the platelet count, and 6126/µL (SD 2824/µL) for the white blood cell (WBC) count.

**Table 1:** Demographics and clinical characteristics of the study participants with COVID-19^a^ (N=5624).

Variables		Value	*P* value for differences in the modified clinical severity score		*P* value for differences in the duration of hospitalization	
**Age (years), n (%)**				<.001		<.001
	0-19	272 (4.8)				
	20-29	1119 (19.9)				
	30-39	564 (10.0)				
	40-49	742 (13.2)				
	50-59	1146 (20.4)				
	60-69	916 (16.3)				
	70-79	545 (9.7)				
	≥80	324 (5.8)				
**Sex, n (%)**				<.001		N/A^b^
	Male	2320 (41.2)				
	Female	3308 (58.8)				
**BMI (kg/m^2^), n (%)**				.002		N/A
	<18.5	260 (5.9)				
	18.5-22.9	1867 (42.2)				
	23.0-24.9	1039 (23.5)				
	≥25	260 (5.9)				
**Systolic blood pressure (mmHg), n (%)**				<.001		.005
	<140	3550 (64.7)				
	≥140	1936 (35.3)				
**Heart rate (beats/min), n (%)**				.003		N/A
	<60	108 (2.0)				
	60-100	4563 (83.0)				
	≥100	828 (15.1)				
**Body temperature, (°C), n (%)**				<.001		<.001
	<37.5	4699 (84.1)				
	≥37.5	887 (15.9)				
**Fever, n (%)**				<.001		<.001
	No	4319 (76.8)				
	Yes	1305 (23.2)				
**Cough, n (%)**				.06		<.001
	No	3283 (58.4)				
	Yes	2341 (41.6)				
**Sputum, n (%)**				.002		<.001
	No	4005 (71.2)				
	Yes	1619 (28.8)				
**Sore throat, n (%)**				<.001		N/A
	No	4743 (84.3)				
	Yes	881 (15.7)				
**Runny nose or rhinorrhea, n (%)**				<.001		N/A
	No	5003 (89.0)				
	Yes	621 (11.0)				
**Muscle aches or myalgia, n (%)**				N/A		.001
	No	4698 (83.5)				
	Yes	926 (16.5)				
**Fatigue or malaise, n (%)**				<.001		.09
	No	5390 (95.8)				
	Yes	234 (4.2)				
**Shortness of breath or dyspnea, n (%)**				<.001		<.001
	No	4958 (88.2)				
	Yes	666 (11.8)				
**Headache, n (%)**				<.001		N/A
	No	4657 (82.8)				
	Yes	967 (17.2)				
**Altered consciousness or confusion, n (%)**				<.001		.04
	No	5589 (99.4)				
	Yes	35 (0.6)				
**Vomiting or nausea, n (%)**				<.001		<.001
	No	5380 (95.7)				
	Yes	244 (4.3)				
**Diabetes mellitus, n (%)**				<.001		<.001
	No	4934 (87.7)				
	Yes	691 (12.3)				
**Hypertension, n (%)**				<.001		<.001
	No	4424 (78.6)				
	Yes	1201 (21.4)				
**Heart failure, n (%)**				<.001		.03
	No	5566 (99.0)				
	Yes	59 (1.0)				
**Chronic cardiovascular disease (except heart failure), n (%)**				<.001		.006
	No	5430 (96.8)				
	Yes	179 (3.2)				
**Asthma, n (%)**				.003		N/A
	No	5497 (97.7)				
	Yes	128 (2.3)				
**Chronic obstructive pulmonary disease, n (%)**				<.001		.02
	No	5585 (99.3)				
	Yes	40 (0.7)				
**Chronic kidney disease, n (%)**				<.001		N/A
	No	5570 (99.0)				
	Yes	55 (1.0)				
**Cancer, n (%)**				<.001		.07
	No	5479 (97.4)				
	Yes	145 (2.6)				
**Chronic liver disease, n (%)**				.004		N/A
	No	5219 (98.4)				
	Yes	83 (1.6)				
**Dementia, n (%)**				<.001		.002
	No	5075 (95.8)				
	Yes	38 (0.7)				
Hemoglobin (g/dL), mean (SD)		13.3 (1.8)	<.001		<.001	
Hematocrit (%), mean (SD)		39.2 (5.0)	<.001		<.001	
Lymphocytes (%), mean (SD)		29.1 (11.7)	<.001		<.001	
Platelet count (/μL), mean (SD)		236734 (82921)	<.001		<.001	
White blood cell count (/μL), mean (SD)		6126 (2824)	<.001		N/A	

^a^*P* values were obtained through Pearson correlation analysis for the modified clinical severity score and with the Cox proportional hazards model for the duration of hospitalization.

^b^N/A: not applicable.

### The Severity of COVID-19

Based on the severity of COVID-19, determined from the mCSS, patients were divided into four levels: mild (n=4455, 79.5%), moderate (n=330, 5.9%), severe (n=512, 9.1%), and critical (n=304, 5.4%). Among patients aged >60 years, 1157 (64.8%) 1567 (87.8%) belonged to the severe and critical levels, respectively. Specifically, patients in the severe and critical levels and aged ≥60 years accounted for 135 (26.4%) and 58 (19.1%), respectively, of the mCSS cohort. Patients in the severe and critical levels and aged ≥70 years accounted for 125 (24.4%) and 89 (29.3%), and those aged ≥80 years accounted for 72 (14.1%) and 120 (39.5%), respectively. Furthermore, with respect to the duration of hospitalization, patients aged >60 years were more frequently found in the long-term treatment group than in the short-term treatment group.

### Univariate Analysis

Table 2 shows the association between the 30 key prediction markers and the mCSS, determined through univariate analysis at a 5% significance level. Patients with an older age; high BMI; SBP of ≥140 mmHg; high heart rate; body temperature of ≥37.5°C; 6 subjective clinical findings including fever, sputum, fatigue or malaise, SOB, ACC, and vomiting or nausea (VN); or 10 comorbidities including DM, HTN, HF, chronic cardiovascular disease (CCD), asthma, COPD, chronic kidney disease, cancer, chronic liver disease, and dementia were likely to have a higher risk of severe disease. Men were found to be at a higher risk of having a high mCSS than women (*P*<.001). Furthermore, patients with a high WBC count or low values of 4 laboratory findings (hemoglobin, hematocrit, lymphocytes, and platelets) tended to be at a higher the risk of severe disease (*P*<.001).

**Table 2:** Significant markers associated with the modified clinical severity score of the study participants with COVID-19^a^.

Variable			*t* test (or ANOVA)				Cochran–Armitage trend test				Pearson correlation analysis				Spearman correlation analysis				Kendall rank correlation analysis		
			*t* (or *F*)		*P* value		*T*		*P* value		*r*		*P* value		*ρ*		*P* value		*T*		*P* value
**Qualitative**																					
	Age	N/A^b^		<.001		N/A		N/A		0.41		<.001		0.38		<.001		0.32		<.001	
	Sex	–0.08		<.001		(1)^c^		N/A		–0.05		<.001		–0.03		.01		–0.03		.01	
	BMI	N/A		<.001		N/A		N/A		0.05 (.002)		.002		0.04		.007		0.04		.007	
	Systolic blood pressure	0.11		<.001		N/A		<.001		0.06 (*P*<.001)		<.001		0.05		<.001		0.05		<.001	
	Heart rate	N/A		<.001		N/A		N/A		0.04		.003		0.03 (.03)		N/A		0.03		.03	
	Temperature	0.43		<.001		N/A		<.001		0.18		<.001		0.18		<.001		0.18		<.001	
	Fever	0.39		<.001		N/A		<.001		0.19		<.001		0.19		<.001		0.19		<.001	
	Sputum	0.08		.002		N/A		<.001		0.04		.002		0.04		.006		0.04		.006	
	Sore throat	–0.17		<.001		1		N/A		–0.07		<.001		–0.06		<.001		–0.06		<.001	
	Runny nose or rhinorrhea	–0.19		<.001		1		N/A		–0.07		<.001		–0.07		<.001		–0.06		<.001	
	Fatigue or malaise	0.24		<.001		N/A		<.001		0.06		<.001		0.05		<.001		0.05		<.001	
	Shortness of breath	0.95		<.001		N/A		<.001		0.36		<.001		0.31		<.001		0.30		<.001	
	Headache	–0.12		<.001		1		N/A		–0.05		<.001		–0.05		<.001		–0.05		<.001	
	Altered consciousness or confusion	1.98		<.001		N/A		<.001		0.18		<.001		0.14		<.001		0.14		<.001	
	Vomiting or nausea	0.26		<.001		N/A		<.001		0.06		<.001		0.06		<.001		0.06		<.001	
	Diabetes mellitus	0.56		<.001		N/A		<.001		0.21		<.001		0.19		<.001		0.19		<.001	
	Hypertension	0.57		<.001		N/A		<.001		0.27		<.001		0.25		<.001		0.24		<.001	
	Heart failure	1.22		<.001		N/A		<.001		0.14		<.001		0.13		<.001		0.13		<.001	
	Chronic cardiovascular disease	0.6		<.001		N/A		<.001		0.12		<.001		0.12		<.001		0.11		<.001	
	Asthma	0.23		.01		N/A		.001		0.04		.003		0.04		.005		0.04		<.001	
	Chronic obstructive pulmonary disease	0.93		<.001		N/A		<.001		0.09		<.001		0.08		<.001		0.08		<.001	
	Chronic kidney disease	1.19		<.001		N/A		<.001		0.14		<.001		0.12		<.001		0.12		<.001	
	Cancer	0.43		<.001		N/A		<.001		0.08		<.001		0.07		<.001		0.07		<.001	
	Chronic liver disease	0.28		.02		N/A		.002		0.04		.004		0.04		.005		0.04		.005	
	Dementia	1.26		<.001		N/A		<.001		0.29		<.001		0.29		<.001		0.28		<.001	
**Quantitative**																					
	Hemoglobin	N/A		N/A		N/A		N/A		–0.22		<.001		–0.19		<.001		–0.15		<.001	
	Hematocrit	N/A		N/A		N/A		N/A		–0.24		<.001		–0.21		<.001		–0.17		<.001	
	Lymphocytes	N/A		N/A		N/A		N/A		–0.38		<.001		–0.35		<.001		–0.28		<.001	
	Platelets	N/A		N/A		N/A		N/A		–0.19		<.001		–0.22		<.001		–0.17		<.001	
	White blood cells	N/A		N/A		N/A		N/A		0.12		<.001		0.04		.02		0.03		.02	

^a^For each test, a variable with positive coefficient represents the predictor positively associated with an increase in clinical severity.

^b^N/A: not applicable.

^c^Sex: Female=1, Male=0; clinical findings or comorbidities; Yes=1, No=0.

The results of univariate analysis for the duration of hospitalization are shown in Multimedia Appendix 1, Table S4. We identified 20 key prediction markers associated with the duration of hospitalization. Patients with an older age; SBP of ≥140 mmHg; body temperature of ≥37.5°C; 7 subjective clinical findings including fever, cough, sputum, muscle aches or myalgia, SOB, ACC, and VN; 6 comorbidities including DM, HTN, HF, CCD, COPD, and dementia; or low values of blood parameters including hemoglobin, hematocrit, lymphocytes, and platelets tended to have a long duration of hospitalization.

### Development and Evaluation of the Prediction Model

To develop prediction models for mCSS and the duration of hospitalization, we selected multiple markers using AUC-based stepwise selection and the LASSO method. For the application of statistical and machine learning models, we defined three binary response variables by regrouping the 4 levels of mCSS into two levels as follows; (1) y_1_: mild (mCSS=1) vs above moderate (mCSS≥2), (2) y_2_: below moderate (mCSS≤2) vs above severe (mCSS≥3), and (3) y_3_: below severe (mCSS≤3) vs critical (mCSS=4). Table 3 shows the results of variable selection and evaluation for each y. Details regarding the selected variables are provided in Multimedia Appendix 1, Table S5. For each case, we aimed to develop a parsimonious model with higher predictive power. Variables selected through the LASSO method were determined as the final model for each y. For y_1_, predictors including older age, high body temperature, SOB, low lymphocyte value, and low platelet count were selected as risk factors. The prediction model with these 5 predictors had an AUC of ≥0.83 (ie, AUC=0.830, sensitivity=0.710, and specificity=0.843 for the RF model). For y_2_, older age, high body temperature, SOB, low hematocrit and lymphocyte values, and low platelet count were selected as risk factors. The prediction model with these 6 predictors yielded an AUC of ≥0.865 (ie, AUC=0.865, sensitivity=0.772, and specificity=0.842 for the RF model). For y_3_, older age, SOB, high WBC count, low hemoglobin and lymphocyte values, and low platelet count were selected as risk factors. The prediction model with these 6 predictors yielded an AUC of ≥0.933 (ie, AUC=0.933, sensitivity=0.895, and specificity=0.865 for the RF model).

**Figure 3:**
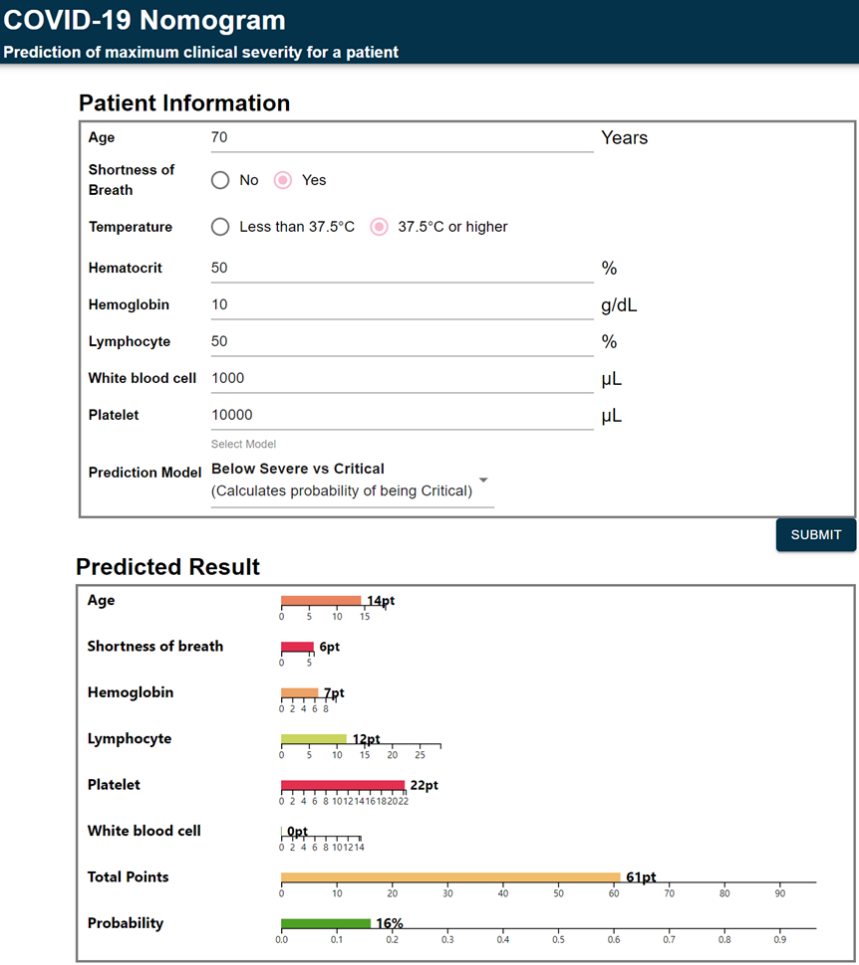
An example of our web-based nomogram.

**Figure 4:**
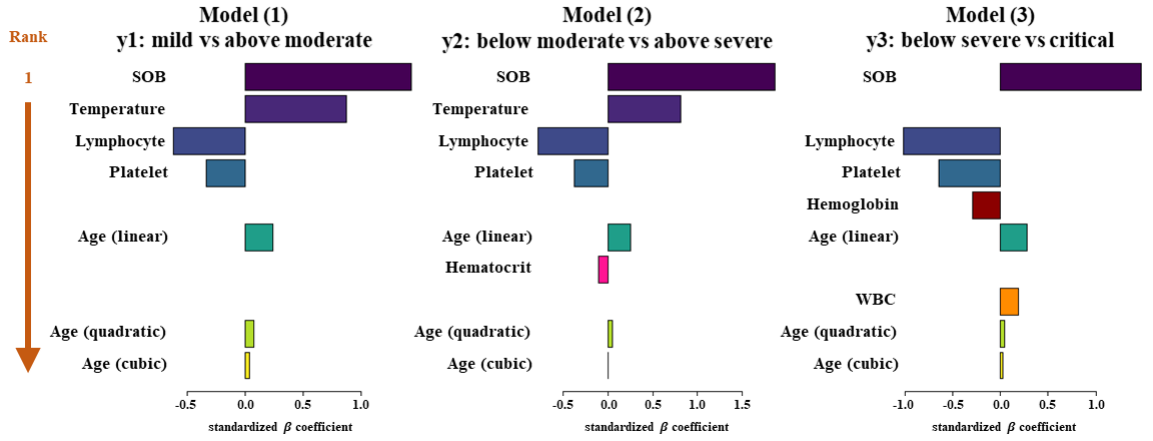
Importance of the predictors. SOB: shortness of breath, WBC: white blood cell.

Based on these 3 prediction models, we developed a prognostic nomogram to predict the mCSS for each patient. The nomogram is available on the internet for clinical use [21]. Figure 3 shows an example of the developed nomogram. The fitted results of the logistic model used to develop the nomogram are shown in Multimedia Appendix 1, Table S6. Based on the standardized *β* coefficients of the fitted results, we ranked the importance of the predictors for each model (Figure 4) [22]. In Figure 4, the x-axis represents the standardized *β* coefficient, and the relative importance of the predictors is shown in descending order for each model. In all 3 prediction models, the SOB ranked first. The temperature selected in the 2 prediction models ranked second in both models, y_1_ and y_2_. In all 3 prediction models, lymphocytes ranked third.

We performed similar analyses for the duration of hospitalization. The results of variable selection and evaluation are summarized in Multimedia Appendix 1, Table S7. The prediction model selected 13 predictors through stepwise selection, including age, hematocrit, cough, FM, platelets, muscle aches or myalgia, dementia, asthma, VN, lymphocytes, WBC count, diarrhea, and body temperature. This model yielded an AUC of ≥0.601. With the LASSO method, only age was selected, and the prediction model yielded a performance of up to 0.571.

**Table 3:** Prediction model and performance for the modified clinical severity score.

Response	Variable selection method	Variables, n	Sample size			Model		Training				Testing		
			Training	Testing			Area under the curve		Sensitivity	Specificity	Area under the curve		Sensitivity	Specificity
y_1_^a^	Stepwise	16	2643	1331	Logistic regression		0.865		0.831	0.745	0.853		0.775	0.797
					Random forest		0.958		0.888	0.888	0.841		0.765	0.792
					Support vector machine		0.87		0.783	0.806	0.856		0.83	0.75
y_1_	Least absolute shrinkage and selection operator	5	2686	1354	Logistic regression		0.85		0.755	0.794	0.847		0.745	0.812
					Random forest		0.905		0.767	0.847	0.83		0.71	0.843
					Support vector machine		0.854		0.77	0.787	0.848		0.739	0.84
y_2_^b^	Stepwise	14	2931	1459	Logistic regression		0.891		0.803	0.807	0.864		0.82	0.765
					Random forest		0.901		0.832	0.85	0.834		0.757	0.837
					Support vector machine		0.887		0.834	0.771	0.854		0.868	0.687
y_2_	Least absolute shrinkage and selection operator	6	2683	1348	Logistic regression		0.881		0.757	0.832	0.877		0.812	0.793
					Random forest		0.943		0.87	0.841	0.865		0.772	0.842
					Support vector machine		0.886		0.835	0.768	0.879		0.812	0.816
y_3_^c^	Stepwise	11	2931	1460	Logistic regression		0.939		0.952	0.795	0.94		0.982	0.766
					Random forest		0.931		0.871	0.925	0.863		0.807	0.91
					Support vector machine		0.933		0.944	0.789	0.935		0.842	0.895
y_3_	Least absolute shrinkage and selection operator	6	2691	1357	Logistic regression		0.923		0.86	0.873	0.944		0.884	0.88
					Random forest		0.991		0.984	0.949	0.933		0.895	0.865
					Support vector machine		0.918		0.812	0.918	0.943		0.874	0.906

^a^y_1_: mild vs above moderate.

^b^y_2_: below oderate vs above severe.

^c^y_3_: below severe vs critical.

## Discussion

### Principal Findings

In this study, we retrospectively assessed the characteristics of 5628 patients with COVID-19 from multiple hospitals in Korea and identified the risk factors for predicting the maximum clinical severity and duration of hospitalization. Older patients aged >60 years accounted for 31.8% of the total, and patients with mild disease accounted for 79.5% of the total. Through univariate analysis for each outcome, we identified 30 risk factors for mCSS and 20 risk factors for the duration of hospitalization. Common risk factors between mCSS and the duration of hospitalization included age, SBP, body temperature, fever, sputum, SOB, ACC, VN, DM, HTN, HF, CCD, COPD, dementia, hemoglobin, hematocrit, lymphocytes, and platelets.

We successfully developed 3 prediction models for mCSS by combining mCSS with 4 levels into 2 levels and developed a web-based nomogram [21] by using these models. Our results indicate that age, body temperature, SOB, lymphopenia, a low hematocrit, low hemoglobin, a low platelet count, and a high WBC count were risk factors positively associated with the maximum clinical severity of COVID-19. These 8 variables have been reported as important predictor variables in the medical literature [23-28]. Specifically, age, shortness of breath, body temperature, lymphocytes, and hemoglobin have been reported as variables for predicting admission to the intensive care unit [23,25], critical illness [24,29], or severe disease [26,27,30]. In particular, Wu et al [26] reported that the severe group had a significantly lower platelet and higher WBC counts than the nonsevere group. Furthermore, Zhang et al [28] reported that hematocrit was significantly lower in the severe group than in the nonsevere group. Our study provides a list of useful risk predictors that can be widely used in a large health care organization during the pandemic.

With an increase in the number of confirmed patients, the number of severely symptomatic patients is also increasing, thus posing a challenge to the management of severe patients during COVID-19 outbreaks. The wide range of outcomes observed, ranging from subpopulations that are mainly asymptomatic to those with markedly high fatality rates, calls for risk stratification. Timely identification of patients at a high risk of developing acute respiratory distress syndrome or multiple organ failure and performing risk stratification management can facilitate more personalized treatment plans and optimized use of medical resources and help prevent further deterioration. To define identify individuals at a high risk of severe disease, the Centers for Disease Control and Prevention defined the following criteria for a high risk of severe disease: age ≥65 years, living in nursing homes, and having at least one underlying comorbidity including chronic lung disease, serious heart conditions, severe obesity, diabetes, chronic kidney disease, liver disease, or an immunocompromised status.

Age and the male gender identified as risk factors of severe COVID-19 in our study have been previously confirmed as risk factors in other countries [31,32]. An elevation in the body temperature is the result of the progression of the infection; hence, if the body temperature is high (≥37.5°C), the prognosis is likely to be poor. In addition, shortness of breath can be considered a symptom that occurs in the course of the disease, since COVID-19 is a type of respiratory disease [33,34]. Among the hematologic abnormalities we observed, we shall consider 2 variables: lymphocytes and platelets. Because lymphopenia and immune dysregulation may impact disease severity, especially because SARS-CoV-2 can directly infect T-lymphocytes, which may be the underlying mechanism of lymphopenia [35]. Regarding the finding of platelet abnormalities, it can be explained that the development of autoimmune antibodies or immune complexes induced by viral infection may play an important role in inducing thrombocytopenia. In addition, SARS-CoV-2 can also directly infect hematopoietic stem or progenitor cells, megakaryocytes, and platelets to inhibit growth and induce apoptosis; furthermore, increased platelet consumption or decreased platelet production in damaged lungs is a potential alternative mechanism that may contribute to thrombocytopenia in severe critical pulmonary conditions [36].

### Limitations

Wynants et al [34] reviewed 50 COVID-19 prediction models and reported that most of the models have a high risk of bias when evaluated with the prediction model risk of bias assessment tool [37]. They found that 2 common causes of bias in prediction models for COVID-19 were the lack of external validation and selection bias. Our study also has these limitations. Since the cohort of patients with COVID-19 in this study includes those whose clinical course has not yet been completed and those who may still potentially develop severe disease, there is a chance that discharged patients without any indication of severe disease during hospitalization would later develop severe disease outside of hospital. In addition, our model was not validated with an external cohort (including foreign data), even though we divided the cohort into a training and testing set to evaluate the predictive power of the developed models. This limitation is mainly due to the limited research environment and the time provided by KDCA to prevent data leakage. Another study limitation is that the data did not include smoking status, which is a very important aspect of an individual's lifestyle, and medication history, especially their history of taking corticosteroids, was not identified. This is an important factor that is closely associated with the exacerbation of the clinical course of COVID-19. The KDCA did not provide information on the smoking status of these individuals because these data were largely missing. In the future, it is expected that more variables from a larger set of patients with COVID-19 be included in the data set to increase the accuracy of the analysis [38,39].

Recently, these prediction tools have been presented in various ways worldwide, but variables with predictive power are identified slightly differently depending on the characteristics of the study population, including nationality and race. In addition, these prediction models can be updated in the current situation where the number of patients continues to rise. Therefore, to develop a model with higher predictive power, it is necessary to constantly compare and validate the results of various studies.

### Conclusions

In this study, we developed models that predict the clinical severity of patients with COVID-19. Compared to previous studies that focused on predicting admission to the intensive care unit [23,25], critical illness [24,29], or severe disease [26,27,30], our model used the largest cohort and showed higher performances, even with a limited number of laboratory variables. Specifically, in the case of the model for predicting the critical group, the predictive power was >0.93. Furthermore, we developed a web-based nomogram [21] that can be easily applied visually.

These models are expected to be used as decision supporting tools at the initial stage of treatment; that is, they can be used to predict patients who might need intensive care owing to deterioration among most patients hospitalized with mild or asymptomatic conditions. They can also help hospitals that manage in-patients acquire and use facilities such as negative pressure beds, mechanical ventilation systems, and extracorporeal membrane oxygenation equipment that must be provided to patients with severe symptoms. If further validated through a prospective study, our prediction model might serve for both rationing decisions at health care levels and selecting patients for randomized controlled trials on new treatment options.

## Appendix

Multimedia Appendix 1 Supplementary tables.

## Abbreviations

ACC: altered consciousness or confusion
AUC: area under the receiver operating characteristic curve
CCD: chronic cardiovascular disease
COPD: chronic obstructive pulmonary disease
CoxPH: Cox proportional hazards
CSS: clinical severity score
DBP: diastolic blood pressure
DM: diabetes mellitus
HF: heart failure
HTN: hypertension
KDCA: Korea Disease Control and Prevention Agency
LASSO: least absolute shrinkage and selection operator
mCSS: modified CSS
RF: random forest
SBP: systolic blood pressure
SOB: shortness of breath
VN: vomiting or nausea
WBC: white blood cell
