# Supplementary material for: Prediction Models for the Clinical Severity of Patients With COVID-19 in Korea: Retrospective Multicenter Cohort Study
Source: J Med Internet Res. 2021 Apr 16;23(4):e25852. doi: 10.2196/25852 (PMC8054775; doi:10.2196/25852)
Supplement: Multimedia Appendix 1 [file jmir_v23i4e25852_app1.doc]

Appendix 1:

Table S1. Classification and list of variables obtained from patient.

| Classification | # Var^a^ | Variable list |
| --- | --- | --- |
| Demographics | 6 | Age, Sex, Quarantine status, Duration of hospitalization, Pregnancy, and Pregnancy week |
| Physical measurement | 1 | Body mass index (BMI) |
| Initial vital signs | 4 | Systolic blood pressure (SBP), Diastolic blood pressure (DBP), Heart rate, and Body temperature |
| Clinical findings | 12 | Fever, Cough, Sputum production, Sore throat, Runny nose/rhinorrhea (RNR), Muscle aches/myalgia, Fatigue/malaise (FM), Shortness of breath/dyspnea (SOB), Headache, Altered consciousness/confusion (ACC), Vomiting/nausea (VN), and Diarrhea |
| Comorbidities: Underlying diseases | 11 | Diabetes mellitus (DM), Hypertension (HTN), Heart failure (HF), Chronic cardiovascular disease (CCD), Asthma, Chronic obstructive pulmonary disease (COPD), Chronic kidney disease (CKD), Cancer, Chronic liver disease (CLD), Rheumatic/autoimmune disease, and Dementia |
| Disease severity | 2 | Admission to an intensive care unit and Maximum clinical severity score during hospitalization |
| Laboratory results | 5 | Hemoglobin, Hematocrit, Lymphocyte, Platelet, and White blood cell (WBC) |

^a^# Var: the number of variables

Table S2. Definition of the CSS.

| Original CSS with 8 levels | Description | Modified CSS with 4 levels |
| --- | --- | --- |
| 1 | No limit of activity | 1 |
| 2 | Limit of activity but No O2 | 2 |
| 3 | O2 with nasal prong | 3 |
| 4 | O2 with facial mask |
| 5 | Non-invasive ventilation | 4 |
| 6 | Invasive ventilation |
| 7 | Multi-organ failure/ECMO |
| 8 | Death |

Table S3. Demographics and clinical characteristics of the study participants with COVID-19 (Full version).^a^

| Variables | | All patients  (n=5,628) | Modified Clinical Severity Score (n=5,601) | | | | Duration of hospitalization (n=5,387) | |
| --- | --- | --- | --- | --- | --- | --- | --- | --- |
|  |  |  | Mild  (n=4,455; 79.5%) | Moderate  (n=330; 5.9%) | Severe  (n=512; 9.1%) | Critical  (n=304; 5.4%) | Short-term (n=2,712; 50.3%) | Long-term (n=2,675; 49.7%) |
| **Age (year)** | | | | | | | | |
|  | 0-19 | 272 (4.8%) | 253 (5.7%) | 16 (4.8%) | 1 (0.2%) | 1 (0.3%) | 170 (6.3%) | 102 (3.8%) |
|  | 20-29 | 1119(19.9%) | 1026 (23%) | 62 (18.8%) | 20 (3.9%) | 2 (0.7%) | 644 (23.7%) | 475 (17.8%) |
|  | 30-39 | 564 (10%) | 512 (11.5%) | 36 (10.9%) | 11 (2.1%) | 5 (1.6%) | 304 (11.2%) | 258 (9.6%) |
|  | 40-49 | 742 (13.2%) | 660 (14.8%) | 43 (13%) | 34 (6.6%) | 2 (0.7%) | 386 (14.2%) | 354 (13.2%) |
|  | 50-59 | 1146(20.4%) | 950 (21.3%) | 50 (15.2%) | 114 (2.3%) | 27 (8.9%) | 569 (21%) | 562 (21%) |
|  | 60-69 | 916 (16.3%) | 674 (15.1%) | 40 (12.1%) | 135(26.4%) | 58 (19.1%) | 377(13.9%) | 505(18.9%) |
|  | 70-79 | 545 (9.7%) | 299 (6.7%) | 32 (9.7%) | 125(24.4%) | 89 (29.3%) | 198 (7.3%) | 274 (10.2%) |
|  | 80 - | 324 (5.8%) | 81 (1.8%) | 51 (15.5%) | 72 (14.1%) | 120(39.5%) | 64 (2.4%) | 145 (5.4%) |
| **Sex** | | | | | | | | |
| Male | | 2320(41.2%) | 1807(40.6%) | 118 (35.8%) | 224(43.8%) | 161 (53%) | 1112 (41%) | 1081(40.4%) |
| Female | | 3308(58.8%) | 2648(59.4%) | 212 (64.2%) | 288(56.2%) | 143 (47%) | 1600 (59%) | 1594(59.6%) |
| **BMI (**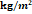**)** | | | | | | | | |
| < 18.5 | | 260 (5.9%) | 195 (5.5%) | 30 (10.4%) | 16 (4%) | 18 (9.9%) | 124 (5.7%) | 120 (5.6%) |
| 18.5-22.9 | | 1867(42.2%) | 1538(43.5%) | 122 (42.4%) | 127(32.1%) | 67 (37%) | 913 (42.3%) | 908 (42.4%) |
| 23-24.9 | | 1039(23.5%) | 829 (23.5%) | 66 (22.9%) | 108(27.3%) | 32 (17.7%) | 522 (24.2%) | 497 (23.2%) |
| ≥25 | | 260 (5.9%) | 195 (5.5%) | 30 (10.4%) | 16 (4%) | 18 (9.9%) | 124 (5.7%) | 120 (5.6%) |
| **SBP (mmHg)** | | | | | | | | |
| < 140 | | 3550(64.7%) | 2851(65.8%) | 217 (66.4%) | 306(59.8%) | 154(53.8%) | 1769 67.1%) | 1663(63.3%) |
| ≥140 | | 1936(35.3%) | 1483(34.2%) | 110 (33.6%) | 206(40.2%) | 132(46.2%) | 867 (32.9%) | 964 (36.7%) |
| **DBP (mmHg)** | | | | | | | | |
| < 90 | | 3921(71.5%) | 3096(71.4%) | 225 (68.8%) | 364(71.1%) | 214(74.8%) | 1881(71.4%) | 1875(71.4%) |
| ≥90 | | 1565(28.5%) | 1238(28.6%) | 102 (31.2%) | 148(28.9%) | 72 (25.2%) | 755(28.6%) | 752 (28.6%) |
| **Heart rate (beats/min)** | | | | | | | | |
| < 60 | | 108 (2%) | 73 (1.7%) | 14 (4.3%) | 15 (2.9%) | 6 (2.1%) | 43 (1.6%) | 59 (2.2%) |
| 60 - 100 | | 4563 (83%) | 3650 (84%) | 272 (83.2%) | 403(78.9%) | 217(75.1%) | 2225(84.1%) | 2171(82.7%) |
| ≥100 | | 828 (15.1%) | 622 (14.3%) | 41 (12.5%) | 93 (18.2%) | 66 (22.8%) | 378 (14.3%) | 396 (15.1%) |
| **Body temperature (°C)** | | | | | | | | |
| < 37.5 | | 4699(84.1%) | 3869(87.4%) | 253 (76.7%) | 354(69.4%) | 198(67.8%) | 2317(86.1%) | 2221(83.3%) |
| ≥37.5 | | 887 (15.9%) | 558 (12.6%) | 77 (23.3%) | 156(30.6%) | 94 (32.2%) | 374(13.9%) | 445 (16.7%) |

^a^Data are presented as the mean ± standard deviation for continuous or number (%) for categorical variables.

Table S3. Continued (Clinical findings).^a^

| Variables | All patients  (n=5,628) | Modified Clinical Severity Score (n=5,601) | | | | Duration of hospitalization (n=5,387) | |
| --- | --- | --- | --- | --- | --- | --- | --- |
|  |  | Mild  (n=4,455; 79.5%) | Moderate  (n=330; 5.9%) | Severe  (n=512; 9.1%) | Critical  (n=304; 5.4%) | Short-term (n=2,712; 50.3%) | Long-term (n=2,675; 49.7%) |
| **Fever** | | | | | | | |
| No | 4319 (76.8%) | 3599(80.9%) | 220 (66.7%) | 296(57.8%) | 180(59.2%) | 2147(79.3%) | 2024(75.7%) |
| Yes | 1305(23.2%) | 852 (19.1%) | 110(33.3%) | 216(42.2%) | 124(40.8%) | 562 (20.7%) | 650 (24.3%) |
| **Cough** | | | | | | | |
| No | 3283(58.4%) | 2622(58.9%) | 215(65.2%) | 239(46.7%) | 190(62.5%) | 1681(62.1%) | 1442(53.9%) |
| Yes | 2341(41.6%) | 1829(41.1%) | 115(34.8%) | 273(53.3%) | 114(37.5%) | 1028(37.9%) | 1232(46.1%) |
| **Sputum production** | | | | | | | |
| No | 4005(71.2%) | 3204 (72%) | 253(76.7%) | 319(62.3%) | 210(69.1%) | 2004 (74%) | 1832(68.5%) |
| Yes | 1619(28.8%) | 1247 (28%) | 77 (23.3%) | 193(37.7%) | 94 (30.9%) | 705 (26%) | 842(31.5%) |
| **Sore throat** | | | | | | | |
| No | 4743(84.3%) | 3714(83.4%) | 276(83.6%) | 446(87.1%) | 289(95.1%) | 2274(83.9%) | 2241(83.8%) |
| Yes | 881 (15.7%) | 737 (16.6%) | 54 (16.4%) | 66 (12.9%) | 15 (4.9%) | 435 (16.1%) | 433 (16.2%) |
| **Runny nose/rhinorrhea** | | | | | | | |
| No | 5003 (89%) | 3916 (88%) | 301(91.2%) | 468(91.4%) | 295 (97%) | 2402(88.7%) | 2366(88.5%) |
| Yes | 621 (11%) | 535 (12%) | 29 (8.8%) | 44 (8.6%) | 9 (3%) | 307(11.3%) | 308 (11.5%) |
| **Muscle aches/myalgia** | | | | | | | |
| No | 4698(83.5%) | 3723(83.6%) | 282(85.6%) | 400(78.1%) | 272(89.5%) | 2300(84.9%) | 2178(81.5%) |
| Yes | 926 (16.5%) | 728 (16.4%) | 48 (14.5%) | 112(21.9%) | 32 (10.5%) | 409 (15.1%) | 496 (18.5%) |
| **Fatigue/malaise** | | | | | | | |
| No | 5390(95.8%) | 4286(96.3%) | 321(97.3%) | 475(92.8%) | 282(92.8%) | 2615(96.5%) | 2551(95.4%) |
| Yes | 234 (4.2%) | 165 (3.7%) | 9(2.7%) | 37 (7.2%) | 22 (7.2%) | 94 (3.5%) | 123 (4.6%) |
| **Shortness of breath/dyspnea** | | | | | | | |
| No | 4958(88.2%) | 4133(92.9%) | 313(94.8%) | 332(64.8%) | 154(50.7%) | 2472(91.3%) | 2358(88.2%) |
| Yes | 666 (11.8%) | 318 (7.1%) | 17 (5.2%) | 180(35.2%) | 150(49.3%) | 237 (8.7%) | 316 (11.8%) |
| **Headache** | | | | | | | |
| No | 4657(82.8%) | 3643(81.8%) | 289(87.6%) | 421(82.2%) | 281(92.4%) | 2247(82.9%) | 2182(81.6%) |
| Yes | 967 (17.2%) | 808 (18.2%) | 41 (12.4%) | 91 (17.8%) | 23 (7.6%) | 462 (17.1%) | 492 (18.4%) |
| **Altered consciousness/confusion** | | | | | | | |
| No | 5589(99.4%) | 4445(99.9%) | 328(99.4%) | 512(100%) | 277(91.1%) | 2707(99.9%) | 2664(99.6%) |
| Yes | 3.5 (0.6%) | 6 (0.6%) | 2 (0.6%) | 0 (0%) | 27 (8.9%) | 2 (0.1%) | 10 (0.4%) |
| **Vomiting/nausea** | | | | | | | |
| No | 5380(95.7%) | 4284(96.2%) | 315(95.5%) | 470(91.8%) | 284(93.4%) | 2619(96.7%) | 2536(94.8%) |
| Yes | 244 (4.3%) | 167 (3.8%) | 15 (4.5%) | 42 (8.2%) | 20 (6.6%) | 90 (3.3%) | 138 (5.2%) |
| **Diarrhea** | | | | | | | |
| No | 5106(90.8%) | 4040(90.8%) | 315(95.5%) | 446(87.1%) | 280(92.1%) | 2486(91.8%) | 2397(89.6%) |
| Yes | 518 (9.2%) | 411 (9.2%) | 15 (4.5%) | 66 (12.9%) | 24 (7.9%) | 223 (8.2%) | 277 (10.4%) |

^a^Data are presented as the mean ± standard deviation for continuous or number (%) for categorical variables.

Table S3. Continued (Comorbidities).^a^

| Variables | All patients  (n=5,628) | Modified Clinical Severity Score (n=5,601) | | | | Duration of hospitalization (n=5,387) | |
| --- | --- | --- | --- | --- | --- | --- | --- |
|  |  | Mild  (n=4,455; 79.5%) | Moderate  (n=330; 5.9%) | Severe  (n=512; 9.1%) | Critical  (n=304; 5.4%) | Short-term (n=2,712; 50.3%) | Long-term (n=2,675; 49.7%) |
| **Diabetes mellitus** | | | | | | | |
| No | 4934(87.7%) | 4037(90.7%) | 286(86.7%) | 397(77.5%) | 190(62.5%) | 2471(91.2%) | 2320(86.8%) |
| Yes | 691 (12.3%) | 315 (9.3%) | 44 (9.3%) | 115(22.5%) | 114(37.5%) | 239(8.8%) | 354 (13.2%) |
| **Hypertension** | | | | | | | |
| No | 4424(78.6%) | 3712(83.4%) | 256(77.6%) | 304(59.4%) | 128(42.1%) | 2260(83.4%) | 2067(77.3%) |
| Yes | 1201(21.4%) | 740 (16.6%) | 74 (22.4%) | 208(40.6%) | 176(57.9%) | 450 (16.6%) | 607 (22.7%) |
| **Heart failure** | | | | | | | |
| No | 5566 (99%) | 4433(99.6%) | 325(98.5%) | 497(97.1%) | 284(93.4%) | 2697(99.5%) | 2646 (99%) |
| Yes | 59 (1%) | 19 (0.4%) | 5 (1.5%) | 15 (2.9%) | 20 (6.6%) | 13 (0.5%) | 28 (1%) |
| **Chronic cardiovascular disease (except heart failure)** | | | | | | | |
| No | 5430(96.8%) | 4342(97.8%) | 313(96.3%) | 473(92.4%) | 275(90.5%) | 2638(97.7%) | 2577(96.6%) |
| Yes | 179 (3.2%) | 99 (2.2%) | 12 (3.7%) | 39 (7.6%) | 29 (9.5%) | 62 (2.3%) | 91 (3.4%) |
| **Asthma** | | | | | | | |
| No | 5497(97.7%) | 4362 (98%) | 322(97.6%) | 495(96.7%) | 291(95.7%) | 2658(98.1%) | 2611(97.6%) |
| Yes | 128 (2.3%) | 90 (2%) | 8 (2.4%) | 17 (3.3%) | 13 (4.3%) | 52 (1.9%) | 63 (2.4%) |
| **Chronic obstructive pulmonary disease** | | | | | | | |
| No | 5585(99.3%) | 4435(99.6%) | 327(99.1%) | 502 (98%) | 294(96.7%) | 2697(99.5%) | 2655(99.3%) |
| Yes | 40 (0.7%) | 17 (0.4%) | 3 (0.9%) | 10 (2%) | 10 (3.3%) | 13 (0.5%) | 19 (0.7%) |
| **Chronic kidney disease** | | | | | | | |
| No | 5570 (99%) | 4434(99.6%) | 325(98.5%) | 498(97.3%) | 286(94.1%) | 2694(99.4%) | 2651(99.1%) |
| Yes | 55 (1%) | 18 (0.4%) | 5 (1.5%) | 14 (2.7%) | 18 (5.9%) | 16 (0.6%) | 23 (0.9%) |
| **Cancer** | | | | | | | |
| No | 5479(97.4%) | 4359(97.9%) | 321(97.3%) | 490(95.7%) | 282(92.8%) | 2655 (98%) | 2605(97.5%) |
| Yes | 145 (2.6%) | 92 (2.1%) | 9 (2.7%) | 22 (4.3%) | 22 (7.2%) | 55 (2%) | 68 (2.5%) |
| **Chronic liver disease** | | | | | | | |
| No | 5219(98.4%) | 4089(98.7%) | 317(98.1%) | 490(97.2%) | 296(97.4%) | 2487(98.5%) | 2498(98.5%) |
| Yes | 83 (1.6%) | 55 (1.3%) | 6 (1.9%) | 14 (2.8%) | 8 (2.6%) | 37 (1.5%) | 39 (1.5%) |
| **Rheumatic/autoimmune disease** | | | | | | | |
| No | 5258(99.3%) | 4111(99.3%) | 321(99.7%) | 498(98.8%) | 301 (99%) | 2507(99.3%) | 2513(99.3%) |
| Yes | 38 (0.7%) | 28 (0.7%) | 1 (0.3%) | 6 (1.2%) | 3 (1%) | 17 (0.7%) | 18 (0.7%) |
| **Dementia** | | | | | | | |
| No | 5075(95.8%) | 4089(98.7%) | 268 (83%) | 463(91.9%) | 228 (75%) | 2477(98.1%) | 2432 (96%) |
| Yes | 38 (0.7%) | 52 (1.3%) | 55 (17%) | 41 (8.1%) | 76 (25%) | 47 (1.9%) | 102 (4%) |

^a^Data are presented as the mean ± standard deviation for continuous or number (%) for categorical variables.

Table S3. Continued (Laboratory results).^a^

| Variables | All patients  (n=5,628) | Modified Clinical Severity Score (n=5,601) | | | | Duration of hospitalization (n=5,387) | |
| --- | --- | --- | --- | --- | --- | --- | --- |
|  |  | Mild  (n=4,455; 79.5%) | Moderate  (n=330; 5.9%) | Severe  (n=512; 9.1%) | Critical  (n=304; 5.4%) | Short-term (n=2,712; 50.3%) | Long-term (n=2,675; 49.7%) |
| **Hemoglobin (g/dL)** | 13.3±1.8 | 13.5±1.6 | 13±1.8 | 12.9±1.8 | 12±2.3 | 13.5±1.7 | 13.2±1.7 |
| **Hematocrit (%)** | 39.2±5 | 39.8±4.5 | 38.5±4.7 | 37.8±5.4 | 35.6±6.7 | 40±4.6 | 39±4.8 |
| **Lymphocyte (%)** | 29.1±11.7 | 31.3±10.6 | 28.7±11 | 23.1±11.3 | 15.6±10.9 | 30.6±11.2 | 29.3±11.1 |
| **Platelet (μL)** | 236734±82921 | 244670±78368 | 224638±96672 | 216657±  90595 | 190389±86811 | 245118±81051 | 234569±82121 |
| **While Blood Cell (μL)** | 6126±2824 | 5990±2446 | 6069±3203 | 6011±2936 | 7834±4925 | 6114±2661 | 5915±2455 |

^a^Data are presented as the mean ± standard deviation for continuous or number (%) for categorical variables.

Table S4. Significant markers associated with the duration of hospitalization including quarantine.^a^

| Type | Variable | HR | β | S.E (β) | *P*-value |
| --- | --- | --- | --- | --- | --- |
| Qualitative | Age | 0.93 (0.91-0.94) | -0.08 | 0.007 | *P*<.001 |
|  | SBP | 0.92 (0.87-0.98) | -0.08 | 0.03 | .005 |
|  | Temperature | 0.85 (0.79-0.91) | -0.16 | 0.038 | *P*<.001 |
|  | Fever | 0.89 (0.83-0.95) | -0.12 | 0.03 | *P*<.001 |
|  | Cough | 0.85 (0.81-0.9) | -0.16 | 0.028 | *P*<.001 |
|  | Sputum production | 0.89 (0.84-0.94) | -0.12 | 0.03 | *P*<.001 |
|  | Muscle aches/myalgia | 0.89 (0.83-0.96) | -0.12 | 0.036 | .001 |
|  | SOB | 0.82 (0.75-0.9) | -0.2 | 0.045 | *P*<.001 |
|  | ACC | 0.55 (0.31-0.97) | -0.6 | 0.289 | .04 |
|  | VN | 0.79 (0.69-0.9) | -0.24 | 0.068 | *P*<.001 |
|  | DM | 0.77 (0.7-0.84) | -0.27 | 0.044 | *P*<.001 |
|  | HTN | 0.8 (0.75-0.86) | -0.22 | 0.034 | *P*<.001 |
|  | HF | 0.72 (0.53-0.98) | -0.33 | 0.157 | .03 |
|  | CCD | 0.8 (0.68-0.93) | -0.23 | 0.082 | .006 |
|  | COPD | 0.66 (0.47-0.94) | -0.41 | 0.178 | .02 |
|  | Dementia | 0.77 (0.66-0.91) | -0.26 | 0.083 | .002 |
| Quantitative | Hemoglobin | 1.06 (1.04-1.08) | 0.06 | 0.01 | *P*<.001 |
|  | Hematocrit | 1.02 (1.02-1.03) | 0.02 | 0.003 | *P*<.001 |
|  | Lymphocyte | 1.01 (1-1.01) | 0.01 | 0.001 | *P*<.001 |
|  | Platelet | 1 (1-1) | 7.00E-07 | 1.90E-07 | *P*<.001 |

^a^HR: hazard ratios; β: coefficients; S.E(β): standard error of coefficients

Table S5. Variable list selected by stepwise and LASSO method for each case of the mCSS.

| Case | Variable selection  method | Selected variable list |
| --- | --- | --- |
| (1) | Stepwise | Age + Lymphocyte + SOB + PLT + Dementia + Temperature + WBC + DM + CCD + Sputum + Sore throat + Heart rate + VN + ACC + CLD + CKD |
| (1) | LASSO | Age + Temperature + SOB + Lymphocyte + PLT |
| (2) | Stepwise | Age + SOB + Temperature + BMI + Sore throat + Sex + Sputum + Cancer + VN + RNR + CKD + Heart failure + HTN + DM |
| (2) | LASSO | Age + Temperature + SOB + Hematocrit + Lymphocyte + PLT |
| (3) | Stepwise | Age + SOB + Temperature + BMI + Sex + DM + Sore throat + CKD + ACC + Asthma + RNR |
| (3) | LASSO | Age + SOB + Hemoglobin + Lymphocyte + PLT + WBC |

Table S6. Fitted results of the logistic model for each max CSS model.^a^

| **(a). Mild vs Above Moderate** | Estimate | Std.Error | *P*-value |
| --- | --- | --- | --- |
| (Intercept) | -2.031 | 0.091 | *P*<.001 |
| Age (Linear) | 0.243 | 0.023 | *P*<.001 |
| Age (Quadratic) | 0.076 | 0.021 | *P*<.001 |
| Age (Cubic) | 0.035 | 0.014 | .01 |
| Temperature | 0.875 | 0.14 | *P*<.001 |
| SOB | 1.434 | 0.145 | *P*<.001 |
| Lymphocyte | -0.616 | 0.065 | *P*<.001 |
| Platelet | -0.339 | 0.06 | *P*<.001 |
| **(b). Below Moderate vs Above Severe** | Estimate | Std.Error | *P*-value |
| (Intercept) | -2.77 | 0.126 | *P*<.001 |
| Age (Linear) | 0.249 | 0.033 | *P*<.001 |
| Age (Quadratic) | 0.044 | 0.028 | .12 |
| Age (Cubic) | 0.002 | 0.017 | .92 |
| Temperature | 0.811 | 0.156 | *P*<.001 |
| SOB | 1.872 | 0.153 | *P*<.001 |
| Hematocrit | -0.106 | 0.065 | .10 |
| Lymphocyte | -0.787 | 0.075 | *P*<.001 |
| Platelet | -0.378 | 0.068 | *P*<.001 |
| **(c). Below Severe vs Critical** | Estimate | Std.Error | *P*-value |
| (Intercept) | -4.565 | 0.309 | *P*<.001 |
| Age (Linear) | 0.277 | 0.083 | *P*<.001 |
| Age (Quadratic) | 0.04 | 0.067 | .55 |
| Age (Cubic) | 0.024 | 0.032 | .46 |
| SOB | 1.47 | 0.201 | *P*<.001 |
| Hemoglobin | -0.286 | 0.093 | .002 |
| Lymphocyte | -1.013 | 0.129 | *P*<.001 |
| Platelet | -0.642 | 0.11 | *P*<.001 |
| WBC | 0.186 | 0.077 | .02 |

^a^For age, 3^rd^ order orthogonal polynomial model was used to fit the curvilinear relationship between age and the modified CSS.

Table S7. Prediction model and performance for the duration of hospitalization including quarantine.

| Variable selection method | # Var^a^ | Sample size | |  | Training | | | Testing | | |
| --- | --- | --- | --- | --- | --- | --- | --- | --- | --- | --- |
|  |  | Training | Testing | Model | AUC | Sen | Spe | AUC | Sen | Spe |
| Stepwise^b^ | 13 | 2538 | 1276 | LR | 0.636 | 0.618 | 0.601 | 0.609 | 0.537 | 0.642 |
|  |  |  |  | RF | 0.706 | 0.574 | 0.734 | 0.601 | 0.573 | 0.601 |
|  |  |  |  | SVM | 0.638 | 0.597 | 0.614 | 0.606 | 0.657 | 0.513 |
| LASSO^c^ | 1 | 3606 | 1781 | LR | 0.581 | 0.568 | 0.545 | 0.571 | 0.331 | 0.773 |
|  |  |  |  | RF | 0.576 | 0.568 | 0.545 | 0.57 | 0.331 | 0.773 |
|  |  |  |  | SVM | 0.419 | 0.568 | 0.545 | 0.429 | 0.331 | 0.773 |

^a^# Var: the number of variables

^b^The variables selected in the stepwise selection were age, hematocrit, cough, FM, platelet, muscle aches/myalgia, dementia, asthma, VN, lymphocyte, WBC, diarrhea, and temperature.

^c^Only age was selected by LASSO method.
